# Attenuating the virulence of the resistant superbug *Staphylococcus aureus* bacteria isolated from neonatal sepsis by ascorbic acid, dexamethasone, and sodium bicarbonate

**DOI:** 10.1186/s12866-022-02684-x

**Published:** 2022-11-09

**Authors:** Moustafa M. Saleh, Nehal Yousef, Shokri M. Shafik, Hisham A. Abbas

**Affiliations:** 1 Microbiology and Immunology Department, Faculty of Pharmacy, Port Said University, Port Fuad, Egypt; 2 Microbiology and Immunology Department, Faculty of Pharmacy, Zagazig University, Zagazig, Egypt

**Keywords:** Ascorbic acid, Dexamethasone, Neonatal sepsis, *S. aureus*, Sodium bicarbonate, Virulence inhibitors

## Abstract

**Background:**

Infections affecting neonates caused by *Staphylococcus aureus* are widespread in healthcare facilities; hence, novel strategies are needed to fight this pathogen. In this study, we aimed to investigate the effectiveness of the FDA-approved medications ascorbic acid, dexamethasone, and sodium bicarbonate to reduce the virulence of the resistant *Staphylococcus aureus* bacteria that causes neonatal sepsis and seek out suitable alternatives to the problem of multi-drug resistance.

**Methods:**

Tested drugs were assessed phenotypically and genotypically for their effects on virulence factors and virulence-encoding genes in *Staphylococcus aureus*. Furthermore, drugs were tested in vivo for their ability to reduce *Staphylococcus aureus* pathogenesis.

**Results:**

Sub-inhibitory concentrations (1/8 MIC) of ascorbic acid, dexamethasone, and sodium bicarbonate reduced the production of *Staphylococcus aureus* virulence factors, including biofilm formation, staphyloxanthin, proteases, and hemolysin production, as well as resistance to oxidative stress. At the molecular level, qRT-PCR was used to assess the relative expression levels of *crtM*, *sigB*, *sarA*, *agrA*, *hla*, *fnbA*, and *icaA* genes regulating virulence factors production and showed a significant reduction in the relative expression levels of all the tested genes.

**Conclusions:**

The current findings reveal that ascorbic acid, dexamethasone, and sodium bicarbonate have strong anti-virulence effects against *Staphylococcus aureus*. Thus, suggesting that they might be used as adjuvants to treat infections caused by *Staphylococcus aureus* in combination with conventional antimicrobials or as alternative therapies.

## Introduction

*Staphylococcus aureus* (*S. aureus*) is a Gram-positive, human, commensal pathogen that colonizes about 30% of the human population [1]. *S. aureus* is a common causative pathogen of severe infections in infants, with symptoms ranging from asymptomatic colonization to skin and soft tissue infections, as well as bacteremia, necrotizing pneumonia, and endocarditis [2]. *S. aureus* is the most common nosocomial pathogen and is heavily associated with high morbidity and mortality [3]. Infections with *S. aureus* are particularly problematic since antibiotic resistance is common in *S. aureus* isolates, with methicillin-resistant *S. aureus* (MRSA) being the most clinically significant [4].

*S. aureus* is the second most prevalent cause of infection in neonatal intensive care unit (NICU) infants with very low birth weights that cause late-onset septicemia [5]. Sepsis caused by MRSA is a life-threatening medical condition that involves systemic inflammation throughout the body [6]. Preterm infants are also at high risk for *S. aureus* colonization, a potential risk factor for subsequent infection [7]. Severe *S. aureus* infections are an important challenge in developing countries [8].

Globally, multidrug-resistant (MDR) bacteria are becoming increasingly resistant to antibiotics [9]. A MDR is an acquired non-susceptibility to at least one agent in three or more antimicrobial categories, while an extensively drug-resistant (XDR) is an acquired non-susceptibility to at least one agent in all but two or fewer antimicrobial categories [10].

*S. aureus* ability to cause infections is linked to several virulence features that enable it to adhere to surfaces, avoid the immune system, and produce damage and toxic consequences to the host [11]. The virulence factors of *S. aureus* are numerous and include, for example, bacterial biofilm formation, hemolysins, and staphyloxanthin production. Furthermore, virulence enzymes such as proteases are commonly related to tissue invasion and disease progression [12, 13].

Virulence factors of *S. aureus* are regulated by several regulatory loci and genes, such as sigma factor (σB) which is encoded by the *sigB* gene, accessory gene regulator (*agr*), and staphylococcal accessory regulator (*sarA*). Also, the *hla* gene is found to encode hemolysins production in *S. aureus*. In addition, the *icaA* and *fnbA* genes play a key role in biofilm formation. Moreover, the initial process in the synthesis of staphyloxanthin is catalyzed by the dehydrosqualene synthase (CrtM) enzyme, which is encoded by the *crtM* gene [14–18].

Drugs that target virulence factors may be able to prevent the depletion of the commensal microbiome. As a result, targeting virulence factors may lessen the impact on resistance development [19]. Many previous researches have reported the repurposing of many FDA-approved drugs as promising anti-virulence agents [20]. A promising technique for finding off-label uses for previously approved drugs is drug repurposing that decreases the risk of treatment failure, especially due to safety concerns. It also reduces the cost, effort, and time involved in drug discovery and evaluation [21].

The majority of drugs used in the Neonatal Intensive Care Unit (NICU) are not approved by the Food and Drug Administration (FDA) for use in newborns. When performing neonatal clinical studies, investigators face a variety of obstacles [22]. So, we aimed to investigate the potential inhibitory effects of the FDA-approved drugs (ascorbic acid, dexamethasone, and sodium bicarbonate) on the *S. aureus* virulence factors by phenotypic, genotypic methods, and in vivo model. Our results might help fighting against lethal infections caused by resistant *S. aureus*.

## Methods

### Media and chemicals

Mueller Hinton agar (MHA), Luria-Bertani (LB) broth, and Tryptone Soya Broth (TSB) were purchased from Oxoid, Hampshire, England. The ascorbic acid was obtained from Memphis pharmaceutical company. EIPICO pharmaceutical company provided the dexamethasone, while Sodium bicarbonate was the product of Otsuka Pharmaceutical Industries, Egypt. Other chemicals were of pharmaceutical-grade.

### Bacterial isolate and its identification

Ten *S. aureus* clinical isolates used in the current study were obtained from the stock culture collection of the Microbiology and Immunology Department, Faculty of Pharmacy, Zagazig University. This isolates were recovered from neonates admitted to Nabrouh Central Hospital Intensive Care Unit, Egypt who had a sepsis.

The identity of the isolates was fully confirmed by using VITEK 2 COMPACT (Biomerieux, France). According to manufacturer’s instructions, the samples were sub-cultured on blood agar and incubated at 37 °C for 24–48 h. A sufficient number of colonies of pure culture were used to suspend the microorganism in a 3.0 ml of a sterile saline test tube. The turbidity of this inoculum was adjusted using normal saline to 0.5 McFarland. The McFarland turbidity was measured using the DensiCHEK Plus equipment. The pure bacterial suspension was added to the device by using the VITEK ID GP identification card for bacterial bio-typing and antibiotic susceptibility testing [23].

### Antibiotic susceptibility testing of the tested clinical isolates

The antibiotic susceptibility testing for the clinical isolates were carried out by minimum inhibitory concentration (MIC) determination using the VITEK 2 COMPACT automated machine. The AST-G592 card was used to perform antimicrobial susceptibility tests for gram-positive bacteria by minimum inhibitory concentration estimation using a kit contains 14 antibiotics including, benzylpenicillin (β-lactams), oxacillin (β-lactams), gentamicin (aminoglycosides), ciprofloxacin (quinolones), moxifloxacin (quinolones), erythromycin (macrolides), clindamycin (lincosamides), teicoplanin (glycopeptides), vancomycin (glycopeptides), tetracycline (tetracyclines), tigecycline (glycylcycline), fusidic acid (fusidanes), rifampicin (ansamycins**)**, and Trimethoprim/sulfamethoxazole (sulfonamides).

### Minimum inhibitory concentration (MIC) determination of the tested drugs

According to The Clinical and Laboratory Standards Institute (CLSI), the agar dilution method was used to assess the MIC of sodium bicarbonate, dexamethasone, and ascorbic acid [24]. In brief, overnight cultures of the tested isolates were diluted with Mueller-Hinton broth to reach the turbidity of the 0.5 McFarland standard to get a final concentration of 10^7^ CFU/ml. Nutrient agar plates with varying concentrations of each drug (0.25, 0.5, 1, 2, 4, 8, 16, 32, and 64 mg/ml) were prepared in addition to control plates without drugs. An aliquot of 100 μl of the suspensions of the tested isolates was inoculated on the plates’ surfaces and incubated at 37 °C overnight. The MIC of sodium bicarbonate, dexamethasone, and ascorbic acid was determined as the lowest concentration that prevented observable bacterial growth.

### Assessment of the impact of sub-MIC of sodium bicarbonate, dexamethasone, and ascorbic acid on *S. aureus* growth

The impact of 1/8 MIC of the tested drugs on the growth of *S. aureus* isolates was assessed by inoculating LB broth with an overnight culture of the tested isolates with and without the presence of the tested drugs and incubated overnight at 37 °C. The effect of 1/8 MIC of the three tested drugs on *S. aureus* isolates growth was determined at 600 nm using a spectrophotometer (Biotek, USA) [25].

For more confirmation to exclude any possible effects of the tested drugs on the growth of the tested bacteria, the growth curve experiment was carried out. Briefly, the isolates were cultured in LB to reach an OD_600_ of 0.2. After that, 100 ml volumes were aliquoted into four 500-ml flasks. A concentrations of 1/8 MIC of the three potential inhibitors ascorbic acid (12.5 mg/ml), dexamethasone (4 mg/ml), and sodium bicarbonate (2 mg/ml), were added to the three cultures in flasks and the bacteria in the fourth flask were kept without addition of any inhibitors as a control. The flasks were incubated at 37 °C with aeration followed by OD_600_ measurements every 30 minutes. One ml of each culture was collected immediately after addition of ascorbic acid, dexamethasone, and sodium bicarbonate (t_0_), respectively. In addition, one ml of each culture was collected at 30, 60, 90, 120, 150, 180, 210, 240, 270, 300, 330 and 360 min and the optical density was measured each time at OD_600_ [26].

### Evaluation of the effect of ascorbic acid, dexamethasone, and sodium bicarbonate on the virulence factors production in *S. aureus* using phenotypic tests

#### Biofilm inhibition assessment

The quantitative assay of biofilm formation was carried out as follows. Briefly, a suspension of the tested isolates in TSB was prepared from overnight culture and adjusted to reach 10^6^ CFU/ml. In a volume of 200 μl of the bacterial suspension was added to the microtiter plate wells in the presence and absence of sub-MIC of the tested drugs and incubated for 48 hours at 37 °C. The TSB was smoothly discarded, and the plates were washed with distilled water to eliminate any planktonic cells, followed by air drying. After 20 minutes of treatment with 200 μl of 99% fixing methanol, the biofilm was dyed for 15 minutes with 200 μl of 1% crystal violet solution. After washing the plate, crystal violet was dissolved in 33% glacial acetic acid, and the absorbance of the solubilized dye was measured at 570 nm using a spectrofluorometer (Biotek, USA) [27].

#### Staphyloxanthin inhibition assay

The tested isolates were cultured overnight and the suspensions were adjusted to 0.5 McFarland then swabbed on the TSA agar plates containing sodium bicarbonate, dexamethasone, and ascorbic acid and control plates. After 2 days of incubation at 37 °C, the cells were harvested and the agar plates’ surfaces were washed and rinsed 3 times with distilled water. The obtained suspensions were centrifuged to collect the cell pellets at 6000 rpm for 15 min. The pellets were mixed with 3 ml 99% methanol and heated in a water bath at 55 °C for 30 min with gentle stirring and then cooled for 10 min and centrifuged again at 6000 rpm for 15 min. The yellow pigment was measured at 450 nm using a spectrofluorometer (Biotek, USA) [28].

#### Total protease inhibition assessment

The modified skim milk method was utilized to measure the total proteases in the presence and absence of the tested drugs. *S. aureus* isolates were grown overnight in LB broth with and without 1/8 MIC of the tested drugs, and supernatants were obtained by centrifugation at 10000 rpm for 10 min. Cell-free supernatants were diluted 10-fold in Tris-HCl buffer (pH 7.4). Next, a volume of 500 μl cell-free supernatant from each of the tested isolates was combined with 1 ml of skim milk solution (1.25% in distilled H_2_O) and incubated for 30 minutes at 37 °C. As a measure of proteolytic activity, the turbidities of assay solutions were assessed at OD_600_ using a spectrofluorometer (Biotek, USA) [29].

#### Hemolysin inhibition assay

The hemolysins assay was evaluated by mixing 600 μl of cell-free supernatants prepared as mentioned in the proteases assay in the presence and absence of 1/8MIC of the tested drugs with 600 μl of fresh 2% v/v defibrinated rabbit blood cells in saline. This mixture was incubated at 37 °C for 2 h and then centrifuged at 10000 rpm for 8 min at 4 °C. Released hemoglobin was measured at 540 nm with a spectrofluorometer and was compared to both a negative and positive control (erythrocytes incubated in LB broth and erythrocytes lysed completely with 0.1% SDS, respectively). The formula [X-B/T-B] × 100 was used to calculate hemolysis percentage, where X represents the tested drug-treated or untreated isolates, B represents the negative control, and T represents the positive control. The hemolysis by the treated cultures was expressed as a percentage compared to the hemolysis by untreated ones [30].

#### Sensitivity to oxidative stress assessment

The ability of ascorbic acid, dexamethasone, and sodium bicarbonate to interfere with *S. aureus* resistance to oxidative stress was examined using the modified disk method. The tested isolates in the presence and absence of the tested drugs was cultured in TSB and incubated overnight. Aliquots of 0.1 ml were put on the surface of TSA plates containing 1/8 MIC of the tested drugs. On the surface of TSA plates, sterile paper discs (6 mm) were inserted and 10 μl of hydrogen peroxide (1.5%) was added. After incubating the plates for 24 hours at 37 °C, the inhibition zones were measured [31].

### RNA extraction and relative gene expression determination using qRT-PCR

The most resistant isolate (isolate 1) was chosen to perform the genotypic experiment as a representative example. *S. aureus* was cultured overnight at 37 °C in TSB with and without 1/8 MIC of sodium bicarbonate, dexamethasone, and ascorbic acid until the bacteria reached the middle log phase (OD_600_ of 0.5–0.6). The pellets were obtained by centrifugation at 6000 g for 15 min, and RNA was extracted using the GeneJET RNA Purification Kit (Thermo Fisher Scientific Inc., Germany) according to manufacture instructions. Reverse transcription followed by qRT-PCR of virulence factors genes *crtM, sigB, sarA, agrA, hla, fnbA, and icaA* was performed followed the protocol described in SensiFAST™ SYBR® Hi-ROX One-Step Kit (Bioline, UK). RT-qPCR analysis was performed using a StepOne RT-PCR thermal cycler (Applied Biosystem, USA) using primers described in Table 1. The housekeeping gene 16S rRNA was used to standardize the relative expression values of each gene. The 2^−∆∆CT^ approach was used to compare the relative gene expression in the treated and untreated isolates [36].

**Table 1:** Primers used in qRT-PCR

Genes	primers	Reference
*crtM*	F / CTGCTAATTCTATGATTGGTTGTGC R / TGGGAATATTATGCAGCTATMGCAG	[32]
*sigB*	F /5-AAG TGA TTC GTA AGG ACG TCT-3 R/ 5-TCG ATA ACT ATA ACC AAA GCC T-3	[32]
*sarA*	F/ TCT TGT TAA TGC ACA ACA ACG TAA R/ TGT TTG CTT CAG TGA TTC GTT T	[33]
*agrA*	F/ GGA GTG ATT TCA ATG GCA CA R/ ATC CAT TTT ACT AAG TCA CCG ATT	[33]
*hla*	F/ GAA AGG TAC CAT TGC TGG TCA R / AAG GCC AGG CTA AAC CAC TT	[33]
*fnbA*	F/ AAC TGC ACA ACC AGC AAA TG R/ TTG AGG TTG TGT CGT TTC CTT	[33]
*ica*	F: CAATACTATTTCGGGTGTCTTCACTCT R: CAAGAAACTGCAATATCTTCGGTAATCAT	[34]
16 s rRNA	F/ 5-TGT CGT GAG ATG TTG GG-3 R / 5-CGA TTC CAG CTT CAT GT-3	[35]

*F* Forward, *R* Reverse

### The mice survival model

The protective activities of ascorbic acid, dexamethasone, and sodium bicarbonate against *S. aureus* pathogenesis were investigated by the mice survival model [37]. The *S. aureus* cultures with or without sub-MIC of the tested drugs were adjusted to 1 × 10^8^ CFU/ml in phosphate-buffered saline (PBS). The study was conducted in six groups, each consisting of 10 albino mice (*Mus musculus*) of similar weight and health characteristics. There are two negative control groups; one group was administered intraperitoneally with 100 μl of sterile PBS and the other group was left un-inoculated. A positive control group was injected with 100 μl of untreated *S. aureus*. The three other groups were injected with 100 μl of overnight bacterial cultures in LB broth in presence of sub-MIC of ascorbic acid, dexamethasone, and sodium bicarbonate, respectively. Three consecutive days were observed to determine the survival rate of experimental mice.

### Statistical analysis

The data in the current study were analyzed with the help of the GraphPad Prism 8 software package. The effect of the tested drugs on *S. aureus* virulence factors was compared using One Way ANOVA at *P* > 0.05 and > 0.001 for significance. The means and standard errors of three biological experiments with three technical replicates were used to calculate the results.

## Results

### Clinical isolates identification

The clinical isolates in the current study were confirmed as *S. aureus* using VITEK 2 COMPACT system (BIOMERIEUX) for bacterial bio-typing and antibiotic susceptibility patterns.

### Antibiotic susceptibility and resistance profile of the tested isolates

The tested isolates showed a high level of resistance against the antibiotics tested. From the 10 isolates, 100% resistance was found against each of benzylpenicillin, and fusidic acid. While the resistance rates were 80% against oxacillin, erythromycin, and vancomycin, and clindamycin (8 isolates), 60% against ciprofloxacin, tiecoplanin, and tetracycline (6 isolates), 50% against trimethoprim/sulfamethoxazole (5 isolates) and 40% against gentamicin, and rifampicin (4 isolates). Tigecycline was the most effective antibiotic with a resistance rate of 10% (one isolate). The complete results of antibiotic susceptibility testing are illustrated in Table 2.

**Table 2:** Antibiotic susceptibility pattern of the tested clinical isolates

Isolates	Isolate1	Isolate 2	Isolate 3	Isolate 4	Isolate 5	Isolate 6	Isolate 7	Isolate 8	Isolate 9	Isolate 10
Antibiotics and their groups										
Benzylpenicillin (β-lactams)	R	R	R	R	R	R	R	R	R	R
Oxacillin (β-lactams)	R	R	R	R	S	R	R	R	R	S
Gentamicin (aminoglycoside)	R	R	S	R	S	R	S	S	S	S
Ciprofloxacin (quinolones)	R	R	S	R	R	R	R	S	S	S
Moxifloxacin (quinolones)	R	R	S	I	R	I	R	S	S	S
Erythromycin (macrolides)	R	R	R	R	R	R	R	R	S	S
Clindamycin (lincosamides)	R	R	R	R	S	R	R	R	R	S
Teicoplanin (glycopeptide)	R	R	R	R	S	R	R	S	S	S
Vancomycin (glycopeptide)	R	R	R	R	R	R	R	I	S	R
Tetracycline (Tetracyclines)	R	S	R	R	S	R	R	R	S	S
Tigecycline	R	S	S	S	S	S	S	S	S	S
Fusidic acid	R	R	R	R	R	R	R	R	R	R
Rifampicin (ansamycins)	R	S	S	R	S	R	R	S	S	S
Trimethoprim/ Sulfamethoxazole (sulphonamides)	S	S	S	R	R	R	R	R	S	S

*R* Resistant, *S* Sensitive, *I* Intermediate

Data from antibiotic sensitivity tests revealed that 20% of the isolates (2 out of 10) were XDR. 80% of the isolates were MDR (8 out of 10).

### Determination of MIC of sodium bicarbonate, dexamethasone, and ascorbic acid

The MIC for the 10 isolates was determined using the broth micro-dilution method. Ascorbic acid, dexamethasone, and sodium bicarbonate inhibited the growth of the tested *S. aureus* isolates at 100 mg/ml, 32 mg/ml, and 16 mg/ml, respectively. The inhibitory activities against *S. aureus* virulence factors were tested at concentrations equivalent to 1/8 MIC of the tested drugs, (2 mg/ml) for sodium bicarbonate, (4 mg/ml) for dexamethasone, and (12.5 mg/ml) for ascorbic acid as other sub-inhibitory concentrations did not show any inhibitory impact on virulence factors production by phenotypic tests.

### The impact of sub-inhibitory concentrations of sodium bicarbonate, dexamethasone, and ascorbic acid on *S. aureus* viable growth

**Fig. 1:**
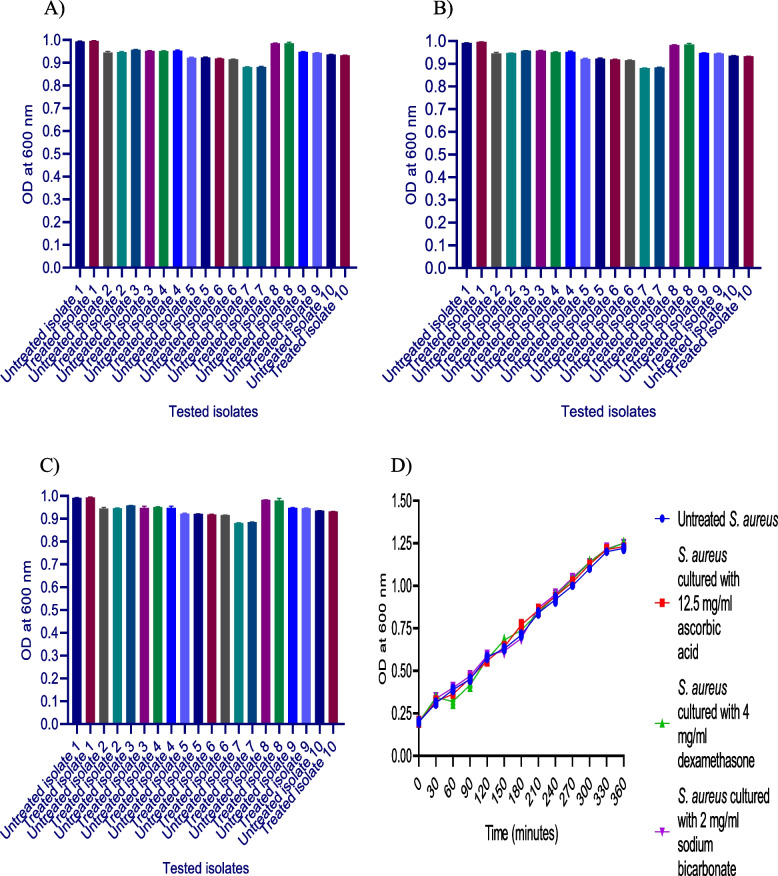
**A**-ascorbic acid, **B**-dexamethasone, and **C**-sodium bicarbonate exhibited no effect on *S. aureus* growth. The OD_600_ of bacteria was measured after culturing overnight in LB broth both in the presence and absence of 1/8 MIC of sodium bicarbonate, dexamethasone, and ascorbic acid. **D**- The growth curve showed no effect of ascorbic acid, dexamethasone, or sodium bicarbonate on *S. aureus* growth. After culturing bacteria in LB broth, OD_600_ was measured every 30 minutes with 1/8 MIC of ascorbic acid, dexamethasone, and sodium bicarbonate when compared to that of untreated bacteria. The tests were performed in triplicate. Values are the averages of three independent experiments. The data shown are the means ± standard errors

The impact of ascorbic acid, dexamethasone, and sodium bicarbonate on *S. aureus* virulence factors may be connected to their inhibitory effect on bacterial growth. To avoid this possibility, the effect of sub-inhibitory concentrations on bacterial growth was evaluated by measuring the optical density at OD_600_ of the overnight cultures with and without the sub-MIC of the three tested drugs. Notably, there was no significant difference observed in the growth of both treated and untreated isolates, proposing that treating *S. aureus* with sub-inhibitory concentrations of the tested drugs has no adverse effect on bacterial growth (Fig. 1A, B, C).

In addition**,** a bacterial growth curve assay was conducted to confirm the non-lethality of the tested drugs. The growth curves of *S. aureus* that was cultured at sub-inhibitory concentrations of ascorbic acid, dexamethasone, and sodium bicarbonate are depicted in (Fig. 1D). Importantly, at 1/8MIC, neither of the tested drugs significantly affected the growth of *S. aureus*.

### Impact of ascorbic acid, dexamethasone, and sodium bicarbonate on *S. aureus* virulence factors production

#### Biofilm inhibition assessment

**Fig. 2:**
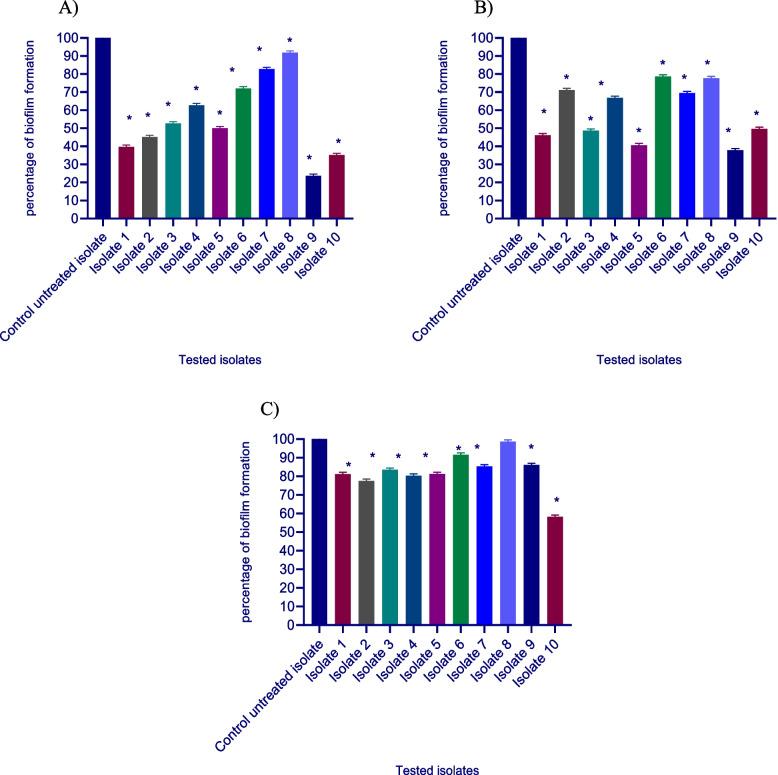
Inhibition of biofilm formation in *S. aureus* by **A**-ascorbic acid, **B-**dexamethasone, and **C-**sodium bicarbonate. A significant reduction of biofilm formation was found with 1/8 MIC of ascorbic acid, dexamethasone, and sodium bicarbonate in untreated isolate compared to treated isolate. The data shown represent the means ± standard errors. One WAY ANOVA test followed by Dunnett’s Multiple Comparison test was used for statistical analysis. *, Significant *P* < 0.05 was considered significant

The crystal violet technique was used to assess the prevention of biofilm development. It’s interesting to note that *S. aureus* ability to produce biofilms was greatly reduced by ascorbic acid, dexamethasone, and sodium bicarbonate. The biofilm formation were reduced by a percentage ranging between (1.35–41.77%), (22.29–63.17%), and (8.13–76.26%) for ascorbic acid, dexamethasone, and sodium bicarbonate, respectively in treated isolates in comparison to untreated isolates (Fig. 2).

### Staphyloxanthin inhibition assay

**Fig. 3:**
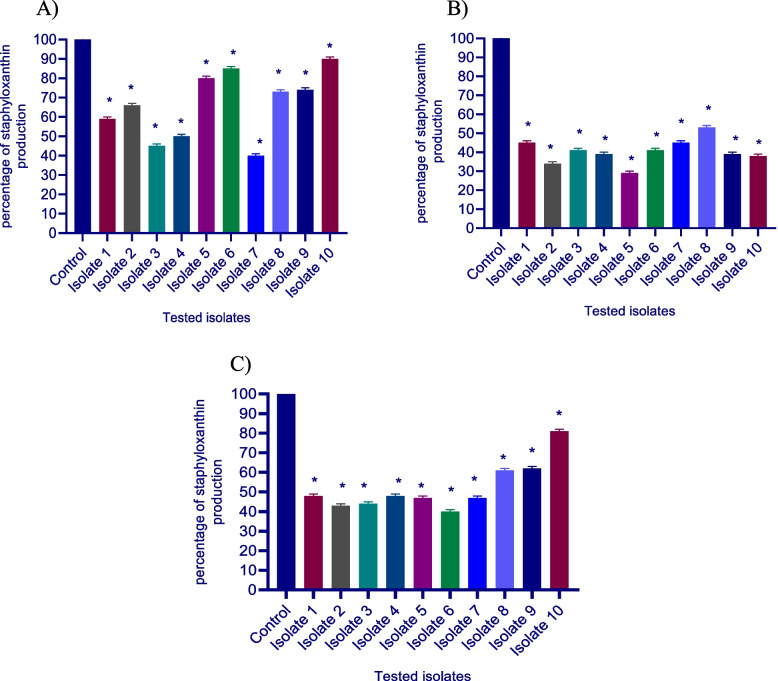
Staphyloxanthin production was significantly reduced in **A-**ascorbic acid, **B-**dexamethasone, and **C-**sodium bicarbonate-treated isolates compared to control untreated bacteria. The absorbance of staphyloxanthin was measured at 560 nm after overnight incubation. The results shown are the means ± standard errors of three biological experiments with three technical replicates each. *, significant *P* < 0.05 (following One Way ANOVA) was considered significant

Ascorbic acid, dexamethasone, and sodium bicarbonate treated isolates displayed a striking decrease in staphyloxanthin production (Fig. 3). The production in treated isolates was reduced to (10–60%) by ascorbic acid (55–71%) by dexamethasone, and, (19–60%) by sodium bicarbonate, respectively in comparison to control untreated isolate.

### Total protease inhibition assessment

**Fig. 4:**
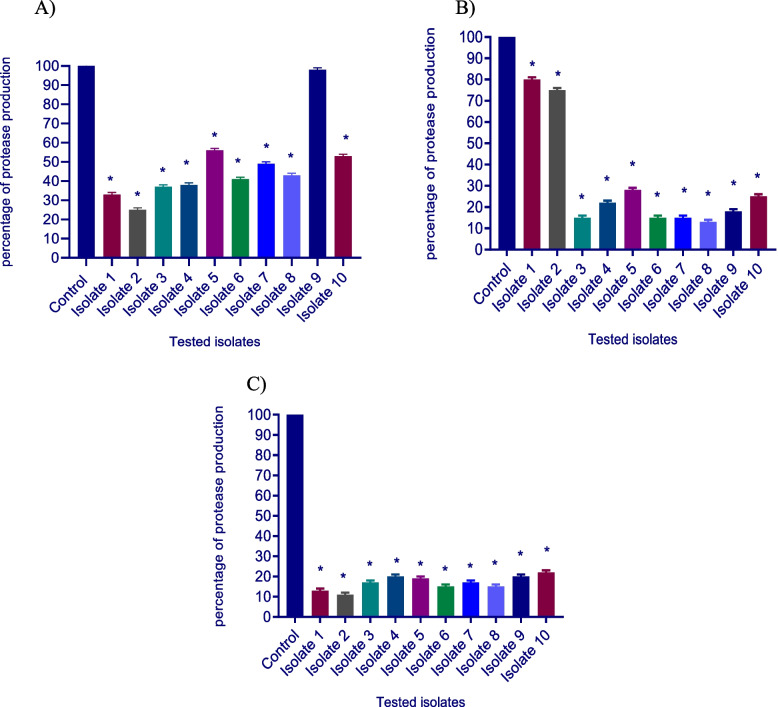
Significant reduction of protease production was found with **A-**ascorbic acid, **B-**dexamethasone, and **C-**sodium bicarbonate-treated isolates. OD_600_ was measured following overnight culturing of bacteria in LB broth in the presence and absence of 1/8 MIC of tested isolates followed by incubation of supernatants with skim milk for 30 min at 37 °C. The data shown are the means ± standard errors of three biological experiments with three technical replicates each. *, significant *P* < 0.05

The proteolytic activity was examined using the modified skim milk assay method with and without the presence of sub-MICs of the tested drugs. The inhibitory effect of proteases activity obtained with ascorbic acid was (44–75%). Dexamethasone, and sodium bicarbonate also showed a significant decrease in proteases activity by (20–87%), and (78–89%) respectively (Fig. 4).

### Hemolysin inhibition assay

**Fig. 5:**
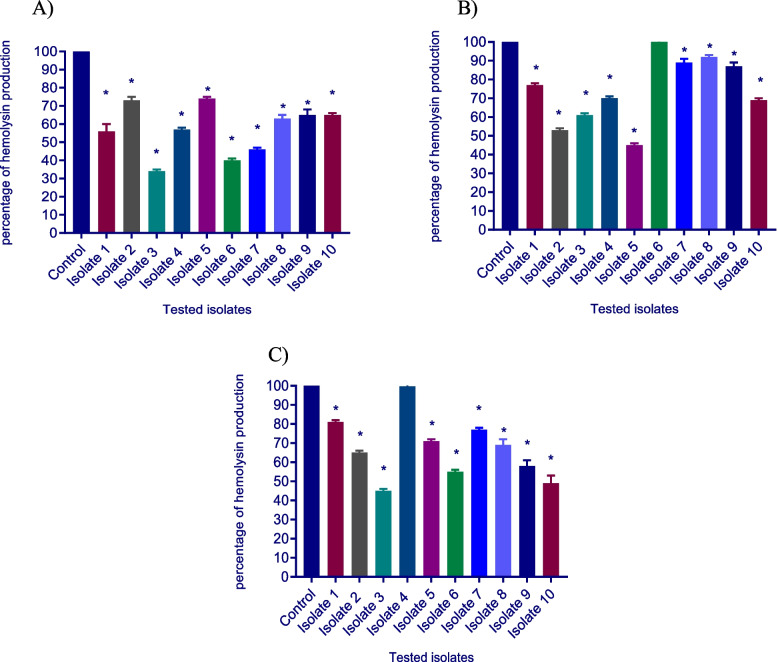
a significant decrease in hemolytic activity was found with **A-**ascorbic acid, **B-**dexamethasone, and **C-**sodium bicarbonate-treated isolates. The hemolytic activity of the drug-free supernatant was considered as 100% hemolysis (control), and the percentage of hemolysis in presence of 1/8 MIC of sodium bicarbonate, dexamethasone, and ascorbic acid were calculated as compared to the control. The data shown are the means ± standard errors of three biological experiments with three technical replicates each. *, significant *P* < 0.05

To assess the tested drugs’ ability to suppress *S. aureus* hemolytic activity*,* the hemolytic activity of untreated and treated isolates was assessed. Notably, ascorbic acid, dexamethasone, and sodium bicarbonate-treated isolates exhibited a significant decrease in hemolysin activity compared to untreated isolate. The inhibition of hemolysin activity under the effect of ascorbic acid, dexamethasone, and sodium bicarbonate was reduced by a percentage ranging between (27–71%), (8–55%), and (19–56%) respectively at 1/8 MIC as shown in Fig. 5.

### Sensitivity to oxidative stress assessment

**Fig. 6:**
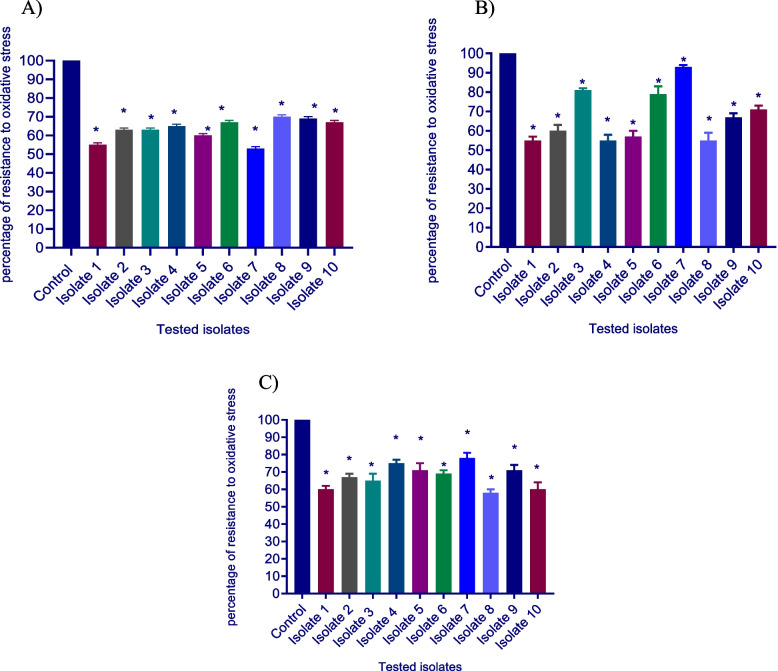
Inhibition of resistance to H_2_O_2_ in *S. aureus* by **A-**ascorbic acid, **B-**dexamethasone, and **C-**sodium bicarbonate. A significant reduction of resistance to H_2_O_2_ was found with 1/8 MIC of ascorbic acid, dexamethasone, and sodium bicarbonate-treated isolates as compared to control untreated bacteria. The data shown are the means ± standard errors of three biological experiments with three technical replicates each. *, significant *P* < 0.05

The impact of ascorbic acid, dexamethasone, and sodium bicarbonate reducing the tolerance of *S. aureus* to oxidative stress was investigated by testing the increasing hydrogen peroxide lethal effect on the growth of *S. aureus* by the tested drugs. Ascorbic acid, dexamethasone, and sodium bicarbonate showed a significant impact in decreasing the tolerance of *S. aureus* tested isolate to oxidative stress by a percentage of (25–40%), (7–45%), and (30–48%), respectively (Fig. 6).

### RT-qPCR estimation of relative gene expression of virulence factors encoding genes in *S. aureus*

To demonstrate that the virulence factors in *S. aureus* may be inhibited by ascorbic acid, dexamethasone, and sodium bicarbonate at the molecular level, we selected the most resistant isolate (isolate 1) for estimating the relative expression of the genes regulating the production of virulence factors in *S. aureus* in treated and untreated isolate using qRT-PCR and was analyzed using the 2^−∆∆Ct^ method.

The relative expression levels of *crtM, sigB, sarA, agrA, hla, fnbA, and icaA* were significantly decreased in ascorbic acid, dexamethasone, and sodium bicarbonate treated isolates in comparison to control untreated isolate. At sub-MIC, ascorbic acid was found to be more potent than sodium bicarbonate and dexamethasone against *S. aureus* virulence factors genes, decreasing expression levels of *crtM, sigB, sarA, agrA, hla, fnbA, and icaA* genes by a percentage ranging between (88.24–95%), in comparison to dexamethasone (30.77–76%), and (65.38–85%), by sodium bicarbonate.

**Fig. 7:**
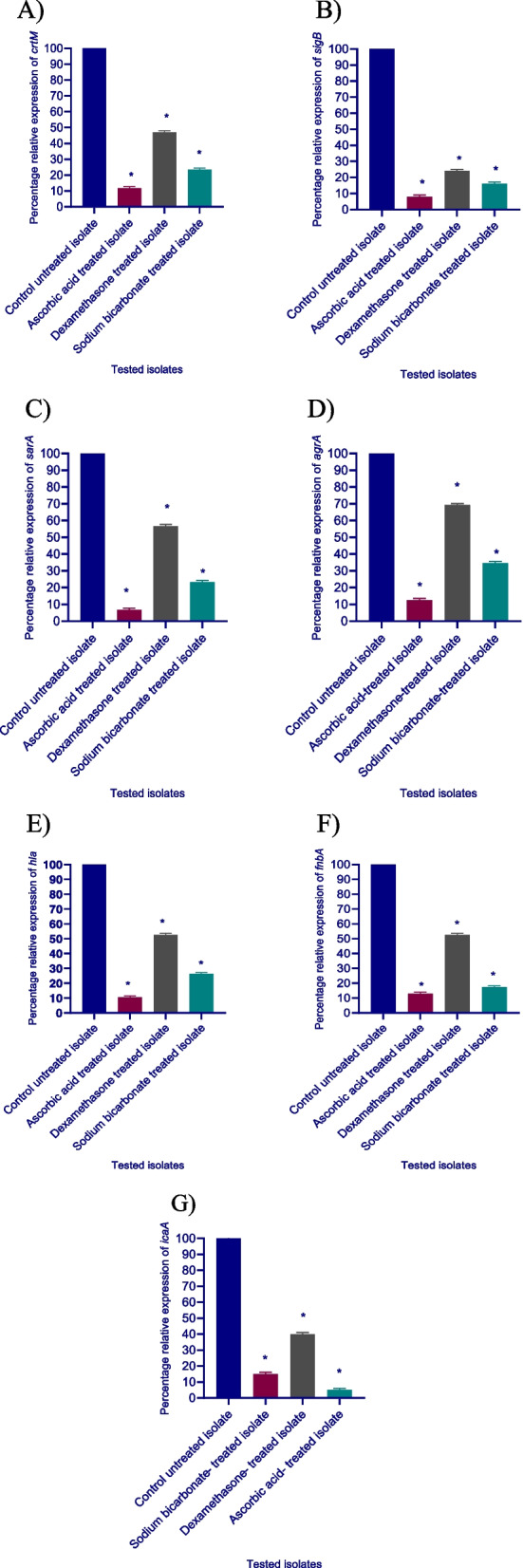
Down-regulation of virulence genes of *S. aureus* by sodium bicarbonate, dexamethasone, and ascorbic acid at 1/8 MIC produced a significant reduction in the expression levels of all the tested virulence genes. **A-** *crtM* gene, **B-** *sigB* gene, **C-** *sarA* gene, **D-** *agrA* gene, **E-** *hla* gene, **F-** *fnbA* gene, **G-** *icaA* gene. The data shown represent the means ± standard errors. One WAY ANOVA test was used for statistical analysis. *, significant P < 0.05

In the current study, ascorbic acid decreased *crtM* relative expression level by 88.24%, while dexamethasone and sodium bicarbonate had 52.94, and 76.47% inhibition effect, respectively; while, *sigB* gene relative expression was suppressed by 84, 76, and 92%in ascorbic acid-, dexamethasone-, and sodium bicarbonate treated-isolates, respectively. In addition, ascorbic acid, dexamethasone, and sodium bicarbonate suppressed the *sarA* gene relative expression by 93.33, 43.33%, and, 76.67% respectively. Also, ascorbic acid inhibited the relative expression level of the *agrA* gene by 88.46% and by 30.77, and 65.38% under the effect of dexamethasone, and, sodium bicarbonate respectively. Moreover, ascorbic acid, dexamethasone, and sodium bicarbonate significantly lowered *hla* relative expression levels by 89.47, 47.37, and73.68%, respectively, while *fnbA* relative expression level was reduced by 86.96% in ascorbic acid-treated isolate, 47.37% in dexamethasone-treated isolate, and 82.61% in sodium bicarbonate-treated isolate. Finally, ascorbic acid, dexamethasone, and sodium bicarbonate reduced the level of relative gene expression of *icaA* by 95, 60, and 85% respectively (Fig. 7).

### The mice survival model

**Fig. 8:**
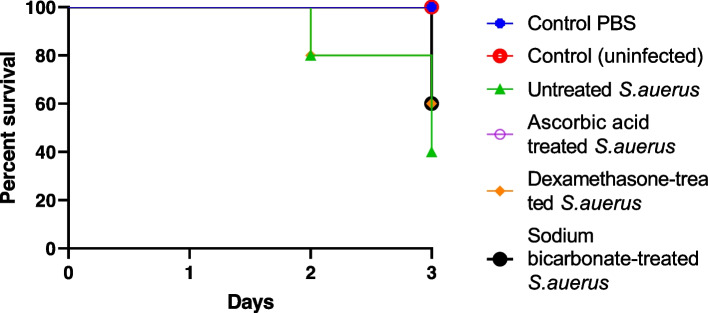
In vivo survival test of *S. aureus*. 6 groups of healthy mice comprising 10 mice each were used. Two negative control groups either injected with sterile PBS or kept uninfected and a positive control group injected with untreated *S. aureus*. 3 test groups were injected with ascorbic acid, dexamethasone, and sodium bicarbonate-treated *S. aureus*. Mice survival in each group was observed every day for 3 days, plotted using the Kaplan-Meier method, GraphPad Prism 8. All mice in the negative control groups survived, while only 40% of mice survived in the positive control group. Ascorbic acid in sub-MIC (12.5 mg/ml) protected all mice, conferring 60% increased protection in comparison with the group inoculated with untreated bacteria. Meanwhile, dexamethasone, and sodium bicarbonate in sub-MIC (2 mg and 4 mg/ml); (6 mice survived) confer 20% increased protection in comparison with the group inoculated with untreated bacteria

The in vivo protective activities of ascorbic acid, dexamethasone, and sodium bicarbonate from *S. aureus* pathogenesis were assessed using six groups of mice. The mice survival was reported for 3 successive days and plotted using the Kaplan-Meier method and significance (*P* < 0.05) was calculated using the Log-rank test, GraphPad Prism 8 (Fig. 8). All mice in negative control groups (uninoculated or PBS injected) totally survived (100%). In the positive control group that was injected with untreated bacteria, only 40% (4 out of 10) of mice were survived. Interestingly, mice injected with ascorbic acid-treated bacteria in sub-MIC showed a significant increase in survival rate of with an increased protection percentage of 60% as compared to those mice injected with untreated bacteria. Additionally, dexamethasone, and sodium bicarbonate also showed a significant rise in survival rate with an increased protection percentage of 20% as compared to those mice injected with untreated bacteria in both drugs.

## Discussion

The antibiotic resistance is one of the greatest threats to public health [38]. The emergence of antibiotic resistance has resulted in the development of *S. aureus* strains that are resistant to nearly all available antibiotics, making treatment a clinical challenge [39]. *S. aureus* infections are a significant clinical burden for newborns around the world, and they were found to be the second most common cause of late-onset sepsis among very low birth weight infants referred to NICUs [7]. Among the most fatal infections for infants is neonatal sepsis, caused mainly by *S. aureus* [40].

To combat the problem of antibiotic resistance, it is now crucial to develop novel treatment approaches, either on their own or in combination with antibiotics; in this context, the term “repurposing” has resurfaced [41]. Several publications have discussed the concept of targeting bacterial virulence rather than viability [42]. The goal of this study is to repurpose the tested drugs in such serious infections caused by MDR *S. aureus*, especially in neonates.

The FDA-approved drugs, ascorbic acid, dexamethasone, and sodium bicarbonate have been extensively used for the treatment of many conventional medical conditions in neonates. Previously, ascorbic acid is also an important physiological antioxidant [43]. Low levels of plasma vitamins C and E are associated with significant hyperbilirubinemia in full-term neonates [44]. Dexamethasone has also been extensively studied in neonatal medicine, where it helps improve pulmonary function and facilitate extubation [45]. In addition, dexamethasone has become widely accepted in standard practice for the postnatal treatment or prevention of chronic lung disease in preterm infants over the last two decades [46]. On the other hand, it was reported that intravenous sodium bicarbonate has been traditionally used to correct metabolic acidosis in neonates [47]. Also, it is used as a therapy designed to prevent azotemia, hypoglycemia, and elevations in serum potassium levels in neonates [48].

Based on previous data that reporting the effectiveness of ascorbic acid, dexamethasone, and sodium bicarbonate as anti-virulence agents in some other pathogens and studies reported the possibility and safety of using them in some health problems in neonates, we tested their effect as anti-virulence agents in 2 XDR, and 8 MDR *S. aureus* isolates from a neonate.

In the current study, the antibacterial activity of the tested drugs ascorbic acid, dexamethasone, sodium bicarbonate, and was determined through MIC assessment. Ascorbic acid was able to inhibit the growth at 100 mg/ml, while dexamethasone inhibited the growth at 32 mg/ml, and Sodium bicarbonate inhibited *S. aureus* growth at 16 mg/ml.

*S. aureus* is a biofilm-forming bacterium that makes therapy difficult. In light of this, focusing on biofilm and other virulence factors may prove to be a viable method for eradicating *S. aureus* infection [49]. *S. aureus* biofilm structure includes polysaccharides and proteins and the bacterial cells compose persistent resistant cells that exhibit multidrug resistance [50]. Other important virulence factors include staphyloxanthin pigment, hemolysins, and proteases that help bacteria to cause infection [35]. Staphyloxanthin is an orange-red triterpenoid carotenoid. It has been suggested that staphyloxanthin can protect *S. aureus* against oxidative stress [51]. Staphyloxanthin has been identified as one of the striking targets of anti-virulent therapy [52]. *S. aureus* also produces extracellular proteases with proposed roles in virulence [53]. *S. aureus* extracellular proteases are recognized to play a key role in pathogenesis; they have been found to compromise the integrity of the airway epithelial barrier, resulting in lung injury [54]. Meanwhile, hemolysins lyse red blood cells. Meanwhile, hemolysins lyse red blood cells, and the most well-known toxin of *S. aureus* is alpha-toxin that can destroy red blood cells and certain types of leukocytes [3].

Five virulence factors in *S. aureus*, including biofilm formation, staphyloxanthin production, proteases, and hemolysins production, and resistance to oxidative stress, were evaluated in the presence of 1/8 MIC (12.5 mg/ml, 4 mg/ml, and 2 mg/ml) of the tested drugs ascorbic acid, dexamethasone, and, sodium bicarbonate respectively. It is important to note that all tested virulence factors were evidently suppressed phenotypically by the tested drugs.

Many previous studies showed results similar to our phenotypic results. For example, in a previous study, ascorbate inhibits proteases and hemolysins’ activities and attenuates the biofilm production of *P. aeruginosa* [55]. Also, in a previous study, ascorbate significantly decreased biofilm production and hemolysins in *Vibrio campbellii* which is similar to our findings [56]. Moreover, in a report following the current results, ascorbic acid exhibited strong dose-dependent bactericidal, anti-biofilm, and virulence-suppressing effects in carbapenem-resistant hypervirulent *Klebsiella pneumonia* [57]. In another report, vitamin C inhibited quorum sensing and other stationary phase regulatory mechanisms that support biofilm development in *E. coli* and *P. aeruginosa* [58].

Moreover, in a previous report, dexamethasone showed anti-biofilm activity against fluconazole-resistant *Candida albicans*, which is in accordance with the present results of the anti-biofilm activity of dexamethasone [59]. In parallel with our results, PYED-1 (pregnadiene-11-hydroxy-16,17-epoxy-3,20-dione-1), which is a synthetic heterocyclic corticosteroid that inhibited the production of *S. maltophilia* biofilms at sub-inhibitory doses [60]. Supporting our results, a previous study showed that a combination of dexamethasone and cloxacillin was more effective than cloxacillin alone in treating bacterial arthritis caused by *S. aureus* in mice [61].

In accordance with the current results, a previous study revealed that sodium bicarbonate impedes the growth and biofilm formation of several pulmonary bacterial pathogens including *S. aureus*, *Streptococcus agalactiae*, *P. aeruginosa*, and *E. coli* [62]. In another study matching our results, delafloxacin efficacy against MDR *S. aureus* is modulated and increased by the effect of bicarbonate [63].

Additionally, the anti-virulence activity of ascorbic acid, dexamethasone, and sodium bicarbonate in the current study against biofilm formation, staphyloxanthin, proteases, hemolysin production, and tolerance to oxidative stress against *S. aureus* is proportional to earlier studies that tested some potential anti-virulence agents. For example, in a previous study, candesartan, domperidone, and miconazole inhibited biofilm formation, proteases, hemolysins, and staphyloxanthin production in *S. aureus* [64]. Furthermore, a recent study showed that hesperidin treatment significantly impedes hemolysin and staphyloxanthin production, which possibly increases the MRSA susceptibility rate to H_2_O_2_ oxidative stress condition which matches to some extent the current results [65]. In another study in accordance with the present study, diclofenac has a remarkable inhibition effect on biofilm formation, hemolysin activity, and staphyloxanthin production against MDR-MRSA [66]. Moreover, glyceryl trinitrate showed significant inhibition of biofilm, staphyloxanthin production, and tolerance to oxidative stress, which is similar to our findings [67]. Also, thymol treatment inhibited staphyloxanthin by 90% and made MRSA cells more susceptible to membrane-targeting antibiotic polymyxin B, which matches the current results to some extent [68].

The molecular understanding of *S. aureus* pathogenesis is critical in the fight against this important human pathogen, since it may aid in the development of new therapeutic techniques [69]. At the molecular level in *S. aureus*, it was found that the *crtM* gene controls the first step in the biosynthesis of staphyloxanthin [70], while the alternative sigma B (*sigB*) gene regulates *S. aureus* response to changing conditions and allows it to adapt to various environments, including those involved in general stress response, virulence, capsule formation, and biofilm formation [14].

In the late stages of growth, the quorum regulator *SarA* of *S. aureus* upregulates the expression of many virulence factors, including biofilm formation, to mediate pathogenesis and immune evasion and has been shown in multiple studies to be a potent regulator of proteases synthesis [71, 72].

Furthermore, *S. aureus’* accessory gene regulator (*agr*) system regulates the expression of virulence factors in response to cell density [73] while the *hla* gene encodes a key virulence factor called hemolysin [74]. *S. aureus* adheres to fibrinogen, elastin, and fibronectin of the host via fibronectin-binding proteins A and B (FnBPA and FnBPB), which are encoded by two genes, *fnbA* and *fnbB*, that are closely related [75].

In this study, the inhibitory activity of sodium bicarbonate, dexamethasone, and ascorbic acid against the regulatory genes *agrA*, *sarA*, *sigB*, and the virulence genes *crtM*, *icaA*, *hla*, and *fnbA* was examined using qRT-PCR. In general, ascorbic acid was found to be the most powerful inhibiting drug against the tested genes, followed by sodium bicarbonate, and finally dexamethasone.

Similarly, hesperidin treatment down-regulated the relative expression of the biofilm-associated gene (*sarA*), the polysaccharide intracellular adhesion regulating gene (*icaA*), the fibronectin-binding protein regulating gene (*fnbA*) and the staphyloxanthin production regulating gene (*crtM*) in *S. aureus* [65]. In proportional with our findings, a previous study that used the three FDA drugs (candesartan, miconazole, and domperidone) reported that the three mentioned drugs decreased the relative expression of *crtM*, *sigB*, *sarA*, *agrA*, *hla*, and *icaA* in *S. aureus*. Additionally, diclofenac had a significant down-regulation effect on the relative expression of virulence regulating genes *sarA*, *agrA*, *hla*, *fnbA*, *icaA*, *sigB*, and *crtM* in *S. aureus* [66]. In another study, parallel with the current results, azan-7 (a novel aza-derivative) significantly reduced *hla* and *agrA* gene expression in MRSA [76]. Moreover, luteolin (a flavonoid of plant origin) and alpha-cyperone (a Chinese medicinal herb) inhibit *hla* and *agrA* gene expression in *S. aureus* [77].

Considering that current data show that *S. aureus* can produce less virulence factors when treated with ascorbic acid, dexamethasone, and sodium bicarbonate, it was crucial to characterize its pathogenesis in vivo. Therefore, mice survival in vivo model was used to evaluate the protective activities of the tested drugs in sub-MIC against *S. aureus* pathogenesis. Importantly, all the tested mice were protected by ascorbic acid, with 60% protection rate more than the mice group inoculated with untreated bacteria. These results are in accordance with our phenotypic and genotypic results. In addition, dexamethasone and sodium bicarbonate were able to give protection rate of 20% more than mice group inoculated with untreated bacteria. Similar to our findings a previous study reported that quercetin was found to be an effective anti-virulence agent and protected mice against lethal pneumonia caused by *S. aureus* [78].

## Conclusions

In light of our results, we could suggest that ascorbic acid, dexamethasone, and sodium bicarbonate represent effective inhibitors of the production of virulence factors in *S. aureus*. This suggests the possibility of using them as an adjuvant therapeutic approach to treat XDR, and MDR *S. aureus* as alternatives or in combination with traditional antibiotics, especially in neonatal sepsis in developing countries. Results such as these can support the repurposing of FDA-approved drugs as a novel strategy to overcome antimicrobial resistance.

## Data Availability

The authors confirm that the data supporting the findings of this study are available within the article.
